# Beyond the Plateau: Repeated Treatment Opportunities Are Associated with Improved Naming in Chronic Post-Stroke Aphasia

**DOI:** 10.3390/brainsci16090905

**Published:** 2026-08-25

**Authors:** Sigfus Kristinsson, Sophia Coolsen, Saeed Ahmadi, Leonardo Bonilha

**Affiliations:** 1 Department of Communication Sciences and Disorders, University of South Carolina, 915 Greene Street, Columbia, SC 29208, USA; scoolsen@email.sc.edu (S.C.); ahmadis@email.sc.edu (S.A.); 2 Department of Neurology, University of South Carolina, Columbia, SC 29208, USA; leonardo.bonilha@uscmed.sc.edu

**Keywords:** aphasia, treatment, naming

## Abstract

**Background/Objectives**: Speech-language treatment improves recovery in chronic post-stroke aphasia, but whether individuals continue to benefit from repeated treatment exposure remains unclear. We tested whether successive treatment episodes were associated with continued naming improvement and whether gains were reduced in later episodes relative to earlier episodes. **Methods**: We analyzed retrospective longitudinal data from 35 participants with post-stroke aphasia who completed at least two aphasia treatment studies. Philadelphia Naming Test scores were obtained before and after each treatment study. The primary analysis used a linear mixed-effects model with fixed effects for timepoint, treatment study, and their interaction, with random intercepts for participants and participant-specific treatment studies. Secondary analyses examined candidate predictors and compared within-treatment with between-treatment change while accounting for elapsed time. **Results**: Naming improved significantly from pre- to post-treatment across studies 1–3 (estimated mean gain = 5.56 points, 95% CI [3.44, 7.67], *p* < 0.001). The timepoint-by-treatment-study interaction was not significant (*p* = 0.384), providing no evidence that gains were attenuated across successive studies. Naming improved faster during treatment than between treatments (adjusted difference = 2.18 points per 30 days, *p* = 0.013). **Conclusions**: Repeated treatment opportunities were associated with continued naming improvement in chronic post-stroke aphasia, supporting a longitudinal view of aphasia rehabilitation.

## 1. Introduction

Aphasia is a common consequence of stroke that affects language abilities, as well as quality of life, psychosocial well-being, and rehabilitation outcomes [1,2]. Anomia is among the most persistent clinical manifestations across aphasia syndromes, and confrontation naming is therefore a central outcome in aphasia treatment research [3]. Confrontation naming accuracy is similarly a clinically meaningful measure of word-retrieval abilities that provides a sensitive and repeatable measure of language recovery [4].

Substantial evidence indicates that speech-language therapy (SLT) can improve language recovery after stroke, and behavioral SLT remains the standard treatment in the clinical management of post-stroke aphasia [5,6]. Meta-analytic, systematic-review, and randomized-trial evidence shows that SLT produces significant gains in language abilities, including in individuals in the chronic stage of recovery [7,8,9,10,11]. However, treatment response is highly variable at the individual level [12]. Recent work focused on explaining this variability suggests that language recovery is shaped by multiple personal- and treatment-related factors, including treatment dose, intensity, and frequency, as well as aphasia severity, age, time post-stroke, and lesion characteristics [10,11,12,13]. The multifactorial nature of treatment-induced aphasia recovery is reflected in contemporary best-practice recommendations, which emphasize access to SLT across the continuum of care, individualized goal-setting, and ongoing reassessment rather than a single, fixed episode of therapy [14].

Despite this progress, the effect of repeated treatment exposure—defined as any form of evidence-based SLT—remains unclear. Most aphasia treatment studies administer a single intervention episode, often with follow-up to assess maintenance, but rarely test whether individuals continue to benefit from successive rounds of SLT. This critical literature gap constrains clinical decision-making: people with aphasia may return for additional therapy months or years after prior treatment because their communication needs change, treatment accessibility changes, or new treatment opportunities become available. Yet clinicians have limited empirical evidence to determine whether additional SLT is likely to support further language recovery or whether prior exposure implies that a practical ceiling has been reached.

A central unresolved question is whether chronic aphasia recovery should be understood as a dynamic process in which individuals can remain responsive to treatment over time. The persistent notion of a chronic recovery plateau has been challenged by longitudinal studies showing that language abilities can change years after stroke, with some individuals improving, some remaining stable, and others declining [12,13,15]. Longitudinal changes in language function have been associated with structural adaptation in residual brain networks, age at stroke onset, and treatment exposure [13,15]. Earlier reviews of the chronic aphasia treatment literature similarly found that time post-onset alone is a poor basis for determining treatment candidacy [16]. Thus, although prior studies have not directly tested whether repeated rounds of treatment support continued language recovery, these findings suggest that aphasia recovery is more dynamic than a strict plateau model would imply.

This question is also important for personalized rehabilitation [12]. Prior work shows that demographic, behavioral, clinical, and neurobiological factors can predict response to aphasia treatment, including differential response to semantic versus phonological treatment [17] and immediate versus long-term recovery [12]. If repeated treatment exposure continues to yield language gains beyond a single treatment episode, personalized aphasia rehabilitation models should move beyond predicting response to a single episode and estimate who is likely to show continued recovery across future treatment opportunities.

The present study addresses these gaps using a retrospective dataset of participants with post-stroke aphasia who completed multiple aphasia treatment studies in the same research environment. The primary aim was to test the hypothesis that more treatment is associated with continued naming improvement, defined as pre- to post-treatment change on the Philadelphia Naming Test (PNT) [4,18] across successive rounds of treatment, and to determine whether treatment-induced recovery is reduced in later relative to earlier rounds of treatment. The secondary aim tested whether participant-level variables predicted PNT change within and across successive rounds of treatment. Additional analyses examined source-study effects, compared within-treatment and between-treatment intervals while accounting for elapsed time, and tested participant-level predictors of total observed naming change across treatment studies.

## 2. Methods

### 2.1. Design and Dataset

This was a retrospective longitudinal analysis of participants with post-stroke aphasia who had participated in aphasia treatment studies in the University of South Carolina Aphasia Lab. Inclusion required completion of at least two out of four source-treatment studies (NCT03416738 [POLAR]; NCT04364854 [SpARc]; NCT01686373 [tDCS]; NIDCD R01 DC009571 [SE]), and the order of source studies varied across participants.

**Figure 1:**
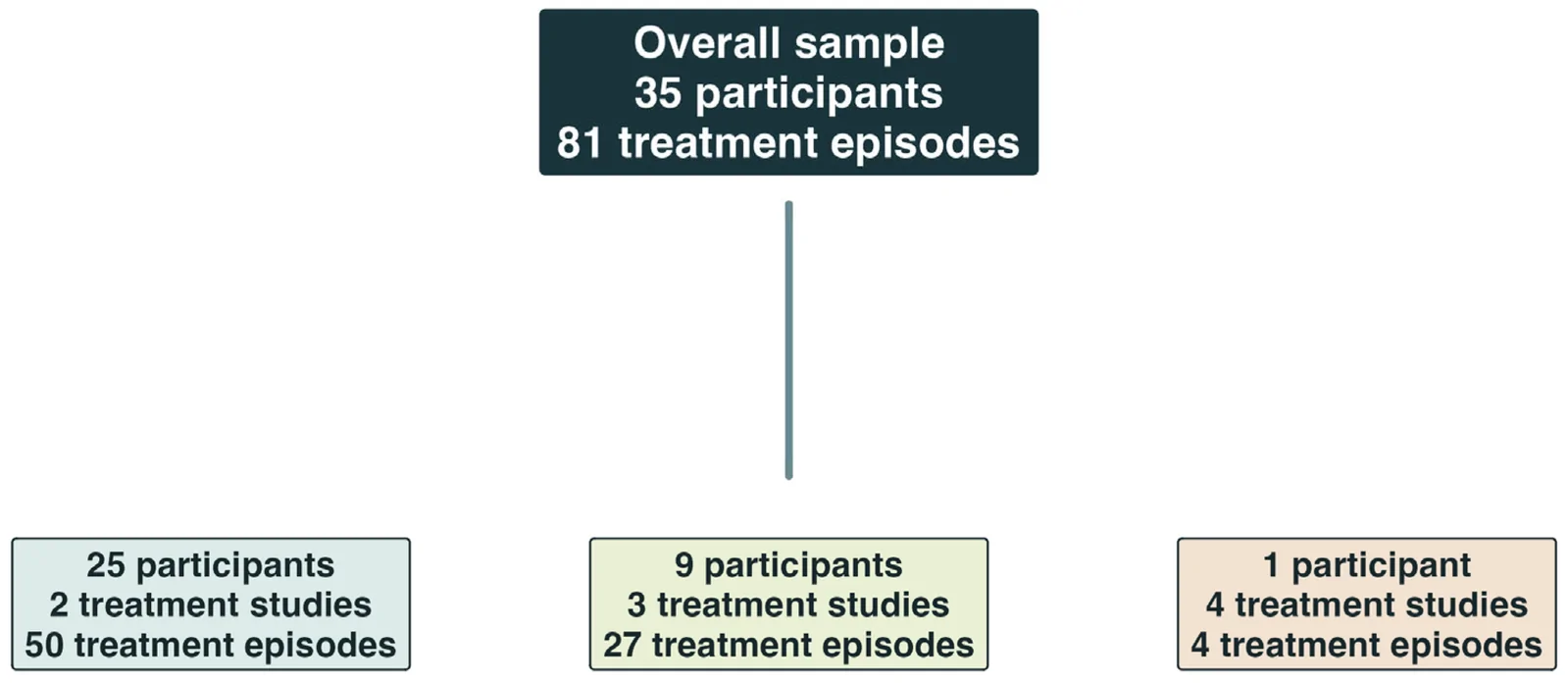
Participant flow and analytic samples for the retrospective dataset.

The four source studies differed in terms of treatment targets, design, and duration but shared a common focus on impairment-focused SLT in chronic post-stroke aphasia. In chronological order, the SE study (NIDCD R01 DC009571) examined neural and behavioral predictors of speech production and perception impairments after left-hemisphere stroke, including the effect of speech entrainment treatment on speech production [19]. The tDCS trial (NCT01686373) was a randomized, sham-controlled Phase II trial testing the effect of transcranial direct current stimulation adjuvant to behavioral SLT on language recovery [20,21]. POLAR (NCT03416738) applied a randomized crossover design focused on examining predictors of response to semantically and phonologically focused anomia treatments [17]. Finally, SpARc (NCT04364854) is a recently completed Phase II trial investigating the effect of treatment dosage on language recovery using speech entrainment therapy [22]. Although each source study was conducted independently of the other studies, the PNT was administered immediately before and after each round of treatments and therefore serves as the primary outcome measure for the analyses reported here. A total of 25 participants completed two studies (50 rounds of treatment), 9 participants completed three studies (27 rounds of treatment), and one participant participated in all four studies (4 rounds of treatment; Figure 1). The distribution of source studies across treatment order is reported in Supplementary Table S1.

### 2.2. Behavioral Measures

The PNT [4,18] was the primary outcome used to assess baseline anomia severity and treatment progress. The PNT is a standardized, computerized confrontation naming assessment composed of 175 low- to high-frequency nouns. None of the items tested on the PNT were included as target treatment items in any of the source studies. Across source studies, graduate research assistants scored and transcribed PNT assessments according to the test manual, under the supervision of American Speech–Language–Hearing Association-certified speech-language pathologists. The administration and scoring protocol remained consistent across studies [23].

The Western Aphasia Battery-Revised (WAB-R) [24] was administered prior to entry into each participant’s first treatment study. For the purpose of examining potential predictors of change in naming performance, aphasia severity was indexed using the WAB-R Aphasia Quotient (WAB-AQ), and the WAB-R was also used to determine participants’ aphasia type [25]. Other candidate predictors were extracted from intake interviews and questionnaires, and included days post-stroke, sex, age at stroke onset, and race.

### 2.3. Aphasia Treatment by Study

#### 2.3.1. SE

Participants completed a 6-week treatment phase with daily 30 min at-home speech production tasks using audiovisual speech entrainment. In these tasks, participants listened to a speaker producing short scripts with visual support (a video showing the speaker’s mouth) and attempted to mimic the model’s speech in real time [19]. The treatment therefore emphasized repeated, externally supported practice of connected speech rather than isolated single-word naming.

#### 2.3.2. tDCS

Participants completed a 3-week computerized SLT program (15 sessions, 45 min each) while receiving either active or sham stimulation. In the active condition, stimulation was delivered at 1 mA for 20 min/session during treatment. The behavioral intervention involved matching pictures depicting common objects with words that were heard (via headphones) and seen (the face of the speaker below the nose shown on a computer screen), providing repeated structured practice intended to improve lexical retrieval [20]. Thus, the behavioral treatment component was consistent across stimulation arms, allowing the study to isolate the added contribution of active neuromodulation relative to sham stimulation.

#### 2.3.3. POLAR

Participants received semantic and phonological treatment in a randomized crossover sequence, with each phase delivered for 3 weeks at 1 h/day, 5 days/week, for a total of 15 h of semantic treatment and 15 h of phonological treatment. The semantic treatment phase was composed of Semantic Feature Analysis, a semantic barrier task, and Verb Network Strengthening Treatment to strengthen lexical-semantic retrieval for nouns and verbs. The phonological treatment phase was composed of Phonological Components Analysis, phonological production practice, and computerized phonological judgment tasks to strengthen word-form retrieval and phonological processing [17]. Treatment was therefore intensive, impairment-focused, and organized around repeated practice with trained lexical items while contrasting semantic versus phonological treatment mechanisms.

#### 2.3.4. SpARc

Participants were randomized to 3, 4.5, or 6 weeks of speech entrainment treatment delivered by telehealth for 1 h/day, 5 days/week, corresponding to 15, 22.5, or 30 total treatment hours. During treatment, participants used an audiovisual computer program to watch and hear a speaker producing scripted speech and then attempted to speak along with the model in real time across repeated trials [22]. The treatment was designed to support connected speech production by externally guiding speech timing, articulation, and fluency through synchronized audiovisual modeling. Three participants completed both the SE and SpARc trials and therefore received two episodes that involved speech entrainment-based treatment, although the studies differed in delivery format and dose structure.

### 2.4. Data Analysis

The primary analysis modeled visit-level PNT scores for treatment studies/rounds 1–3 using a linear mixed-effects model. Fixed effects included timepoint (pre-/post-treatment), treatment rounds (1–3), and their interaction. Treatment round 4 was summarized descriptively but excluded from inferential modeling because only one participant completed all four source studies. The main effect of timepoint tested whether PNT scores changed from pre- to post-treatment, regardless of treatment round. The main effect of treatment round tested whether overall PNT scores differed across the first, second, and third treatment rounds, averaging across pre- and post-treatment assessments. The timepoint-by-treatment-round interaction tested whether the magnitude of pre- to post-treatment change differed across treatment rounds (i.e., whether PNT change differed between later and earlier rounds of treatment). Random intercepts for participant and treatment rounds accounted for repeated observations within individuals and paired pre-/post-treatment observations within each round.

Each candidate predictor was subsequently tested within the primary model. For each predictor, we tested the predictor main effect and the predictor-by-treatment round interaction. This approach preserved the primary model while evaluating whether baseline clinical or demographic characteristics predicted PNT performance overall or differed across treatment order. To reduce sparse cells while preserving clinically interpretable distinctions, aphasia type was collapsed into three groups: Broca’s aphasia, anomic/conduction aphasia, and global/Wernicke’s aphasia. This grouping was guided by traditional aphasia classification frameworks, in which aphasia types are differentiated primarily based on fluency, auditory comprehension, repetition, and naming performance [24,26]. We also tested a study-adjusted version of the primary model to evaluate whether PNT scores differed across source studies and an exploratory study-by-timepoint model to test whether pre-to-post PNT change differed by source study.

We performed several sensitivity analyses to test the robustness of the primary model relative to alternative modeling choices. Specifically, the primary model was refit with the addition of visit-level days post-stroke as a covariate to account for the influence of assessment timeline; treating treatment round as an ordered variable as opposed to a categorical variable to account for potential trends across rounds 1, 2, and 3; excluding participants with unusually large or small overall PNT change scores using the 1.5*IQR rule to ensure the results were not driven by a few extreme outliers; and, with a participant-specific random slope for timepoint to allow greater flexibility in deviations from the average pre- to post-treatment change observed across participants. Moreover, we conducted an exploratory equivalence analysis to test whether naming gains in later rounds of treatment were meaningfully similar to gains in the first treatment round using a +/−5 PNT-point margin.

As an additional interval-level analysis, we compared PNT change within treatment with PNT change between treatment phases. Within-treatment intervals were defined as pre-to-post change within the same round of treatment. Between-treatment intervals were defined as change from the post-treatment assessment of one treatment to the pre-treatment assessment of the next observed round of treatment. The interval-level model predicted adjacent-assessment PNT change from interval type while controlling for days post-stroke at the start of the interval, interval duration, and participant random intercept. Because interval duration differed greatly between within-treatment and between-treatment intervals, a complementary model compared PNT change per 30 days while controlling for days post-stroke at interval start. This analysis was considered descriptive because between-treatment intervals were not experimentally controlled and were much longer than within-treatment intervals.

### 2.5. Secondary Participant-Level Analysis

A secondary analysis examined participant-level change in naming performance across the full period of research participation. For each participant, overall PNT change was calculated as the last observed PNT score minus the first observed PNT score. Separate univariate models were used to examine the association between each candidate predictor and overall PNT change. We then used all-subsets model selection based on the small-sample corrected Akaike information criterion (AICc) to identify the strongest multivariable predictor model. Candidate predictors included days post-stroke, number of treatment rounds completed, sex, age at stroke, race, baseline WAB-AQ, baseline PNT score, and aphasia type.

Our primary hypotheses were tested using prespecified omnibus model effects; therefore, no post hoc multiple-comparison correction was applied to the primary analyses. Secondary and exploratory analyses were interpreted cautiously. Models were fit in R using lme4 and lmerTest, with Satterthwaite degrees of freedom for fixed-effect inference [27,28]. Estimated marginal means and contrasts were computed using emmeans. Figures were generated with ggplot2.

## 3. Results

### 3.1. Participant and Treatment Characteristics

Sample descriptive statistics are shown in Table 1. The sample included 35 participants, most of whom completed two treatment rounds. Distributions of candidate predictor variables are provided in Supplementary Figure S1.

**Figure 2:**
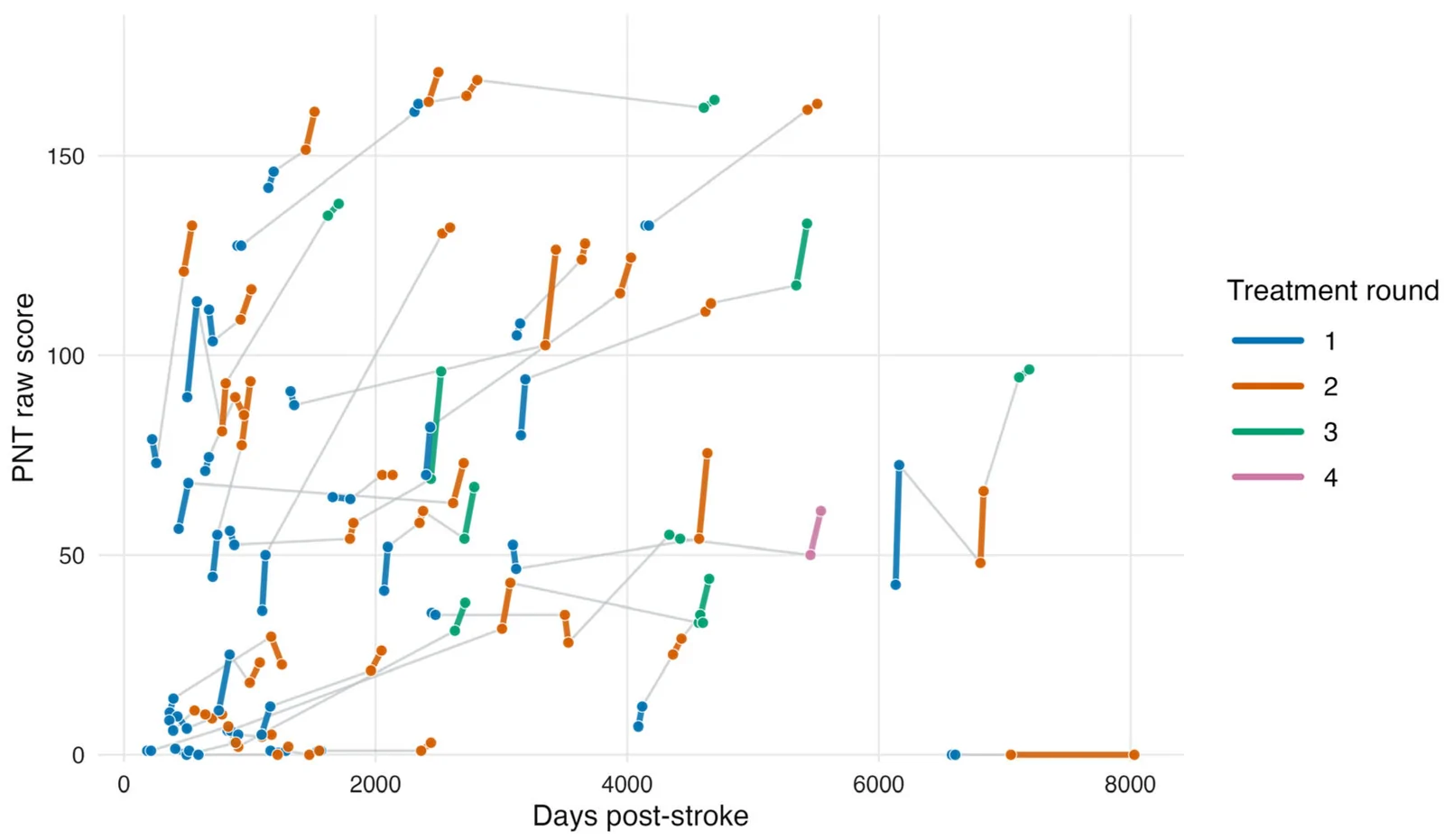
Raw longitudinal PNT score trajectories. Raw PNT scores plotted by days post-stroke for each participant. Thin gray lines connect all available assessments, and colored segments indicate pre- to post-treatment intervals within each treatment round.

Figure 2 displays the raw longitudinal PNT data for all participants included in the primary analysis. Each participant contributed multiple PNT assessments across two to four treatment rounds, with assessments spanning variable intervals after stroke. Although participants differed substantially in initial naming performance and time post-stroke, the raw trajectories show that naming performance frequently increased following treatment periods, with variable trajectories between treatment phases. The raw data also highlight the heterogeneity of recovery: some participants showed large gains across one or more treatment rounds, whereas others showed smaller or more variable changes over time.

### 3.2. Primary Longitudinal Analysis: Is More Treatment Better?

The primary mixed-effects model showed significant pre- to post-treatment improvement in PNT across treatment rounds 1–3. Across treatment rounds, the estimated mean pre- to post-treatment gain was 5.56 points (95% CI [3.44, 7.67], t(82.2) = 5.23, *p* < 0.001). The omnibus timepoint effect was significant (F(1, 79.0) = 28.40, *p* < 0.001).

**Figure 3:**
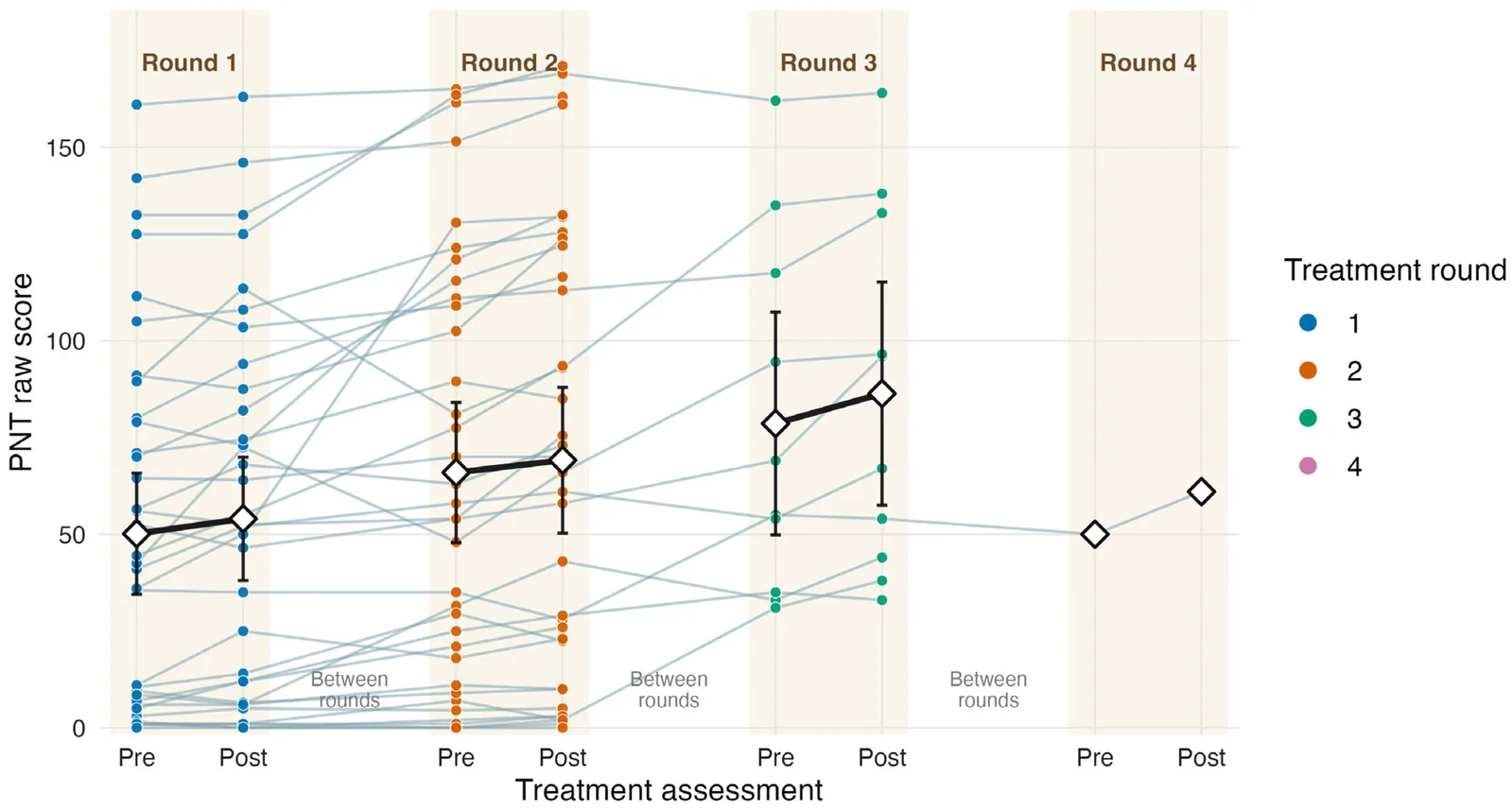
Individual and mean PNT trajectories across treatment rounds. Thin lines follow participants continuously from first to last observed assessment; shaded bands represent within-treatment intervals and intervening spaces represent between-treatment intervals. Mean trajectories are shown in black with 95% confidence intervals.

Estimated pre- to post-treatment contrasts were positive and statistically significant for each of the three rounds of treatment (Table 2): round 1 = 3.86 points, round 2 = 5.07 points, and round 3 = 7.75 points (all *p* < 0.01). The timepoint-by-treatment-round interaction was not significant (F(2, 79.1) = 0.97, *p* = 0.384). Thus, there was no evidence that treatment-induced naming gains were reduced in later rounds relative to earlier rounds of treatment. Figure 3 visualizes individual and mean PNT trajectories across treatment rounds.

Clinical interpretation was supported by responder summaries in the absence of an established minimal clinically significant difference for raw PNT change in a repeated-treatment context. Improvement greater than zero was observed in 17/35 (48.6%) first rounds, 27/34 (79.4%) second rounds, and 8/10 (80.0%) third rounds. Gains of at least 5 PNT points were observed in 11 (31.4%) first rounds, 14 (41.2%) second rounds, and 5 (50.0%) third rounds; gains of at least 10 points were observed in 9 (25.7%), 8 (23.5%), and 4 (40.0%) rounds, respectively (Supplementary Table S2).

Sensitivity analyses supported the primary findings (Supplementary Table S3). The overall pre- to post-treatment effect remained significant after adding visit-level days post-stroke (gain = 5.23), when treatment round was modeled ordinally (gain = 4.87), after excluding one IQR-defined outlier (gain = 5.49), and when allowing a participant-specific random slope for timepoint (gain = 5.53; all *p* < 0.001). The timepoint-by-treatment round interaction was nonsignificant across all sensitivity models. Exploratory equivalence testing using a 5-point margin supported equivalence for round 2 versus round 1 gains but not for round 3 versus round 1 gains; therefore, the primary claim is framed as the absence of statistical evidence for attenuation, not proof that later-round gains are equivalent to earlier-round gains (Supplementary Table S4).

### 3.3. Predictors of Longitudinal PNT Scores

To test the effect of baseline demographic and clinical factors on treated naming recovery, candidate predictors were first added individually to the established primary model and tested as main effects and as interactions with treatment round. Baseline PNT (beta = 46.72, *p* < 0.001), baseline WAB-AQ (beta = 38.64, *p* < 0.001), and aphasia type (anomic/conduction vs. Broca’s: beta = 27.48; global/Wernicke’s vs. Broca’s: beta = −45.06; *p* = 0.012) were significant main-effect predictors of longitudinal PNT scores (Table 3). No significant main effects emerged for days post-stroke, sex, age at stroke, or race, and no significant interactions with treatment round were observed (all *p* > 0.05).

### 3.4. Source Study Effects

To evaluate whether study-specific variability accounted for the primary treatment effects, the primary mixed-effects model was extended to include the main effect of source study and its interaction with timepoint. Source study had a significant main effect on overall PNT scores (F(3, 47.19) = 3.30, *p* = 0.028), indicating that PNT levels differed across source studies. However, the effects of timepoint (F(1, 79.26) = 21.17, *p* < 0.001) and treatment round (F(2, 47.57) = 3.40, *p* = 0.042) remained significant, and the timepoint-by-treatment round interaction remained nonsignificant (F(2, 79.15) = 0.67, *p* = 0.514). The timepoint-by-source study interaction was similarly nonsignificant (F(3, 79.26) = 0.74, *p* = 0.529), indicating that source study did not account for treatment-induced change in naming beyond the effect of cumulative treatment exposure (Supplementary Table S5; Supplementary Figure S2).

### 3.5. Interval-Level Comparison: Within-Treatment Versus Between-Treatment Change

**Figure 4:**
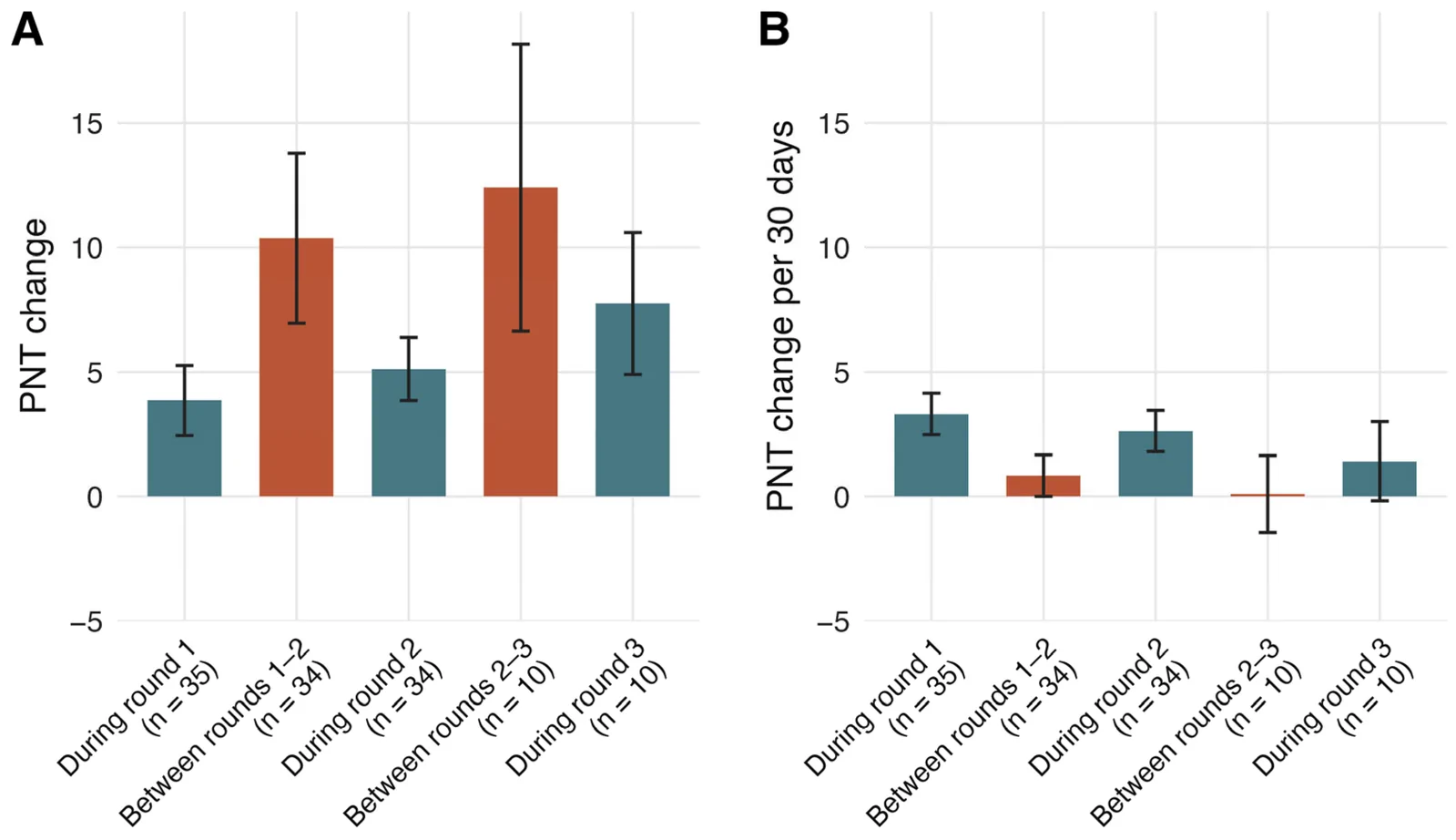
PNT change within- vs. between-treatment intervals. (**A**) shows adjusted absolute PNT change; (**B**) shows adjusted PNT change rate per 30 days.

An interval-level analysis was performed to compare longitudinal PNT change within versus between treatment episodes. Across participants, the dataset included 80 within-treatment episodes and 46 between-treatment intervals. An improvement in PNT scores was observed during both interval types, with a mean change of 4.97 points within-treatment and 10.34 points between treatment episodes. However, since between-treatment intervals were much longer than within-treatment intervals (mean = 769.43 vs. 70.62 days), the rate of PNT change was estimated per 30 days while controlling for days post-stroke at interval start. In this rate-based model, the rate of change was significantly faster during within-treatment intervals than between-treatment intervals: adjusted within-treatment rate = 2.80 PNT points per 30 days, adjusted between-treatment rate = 0.63 points per 30 days; mean difference = 2.18, 95% CI [0.47, 3.88], *p* = 0.013 (Figure 4).

### 3.6. Secondary Participant-Level Analysis: Predictors of Overall PNT Change

**Figure 5:**
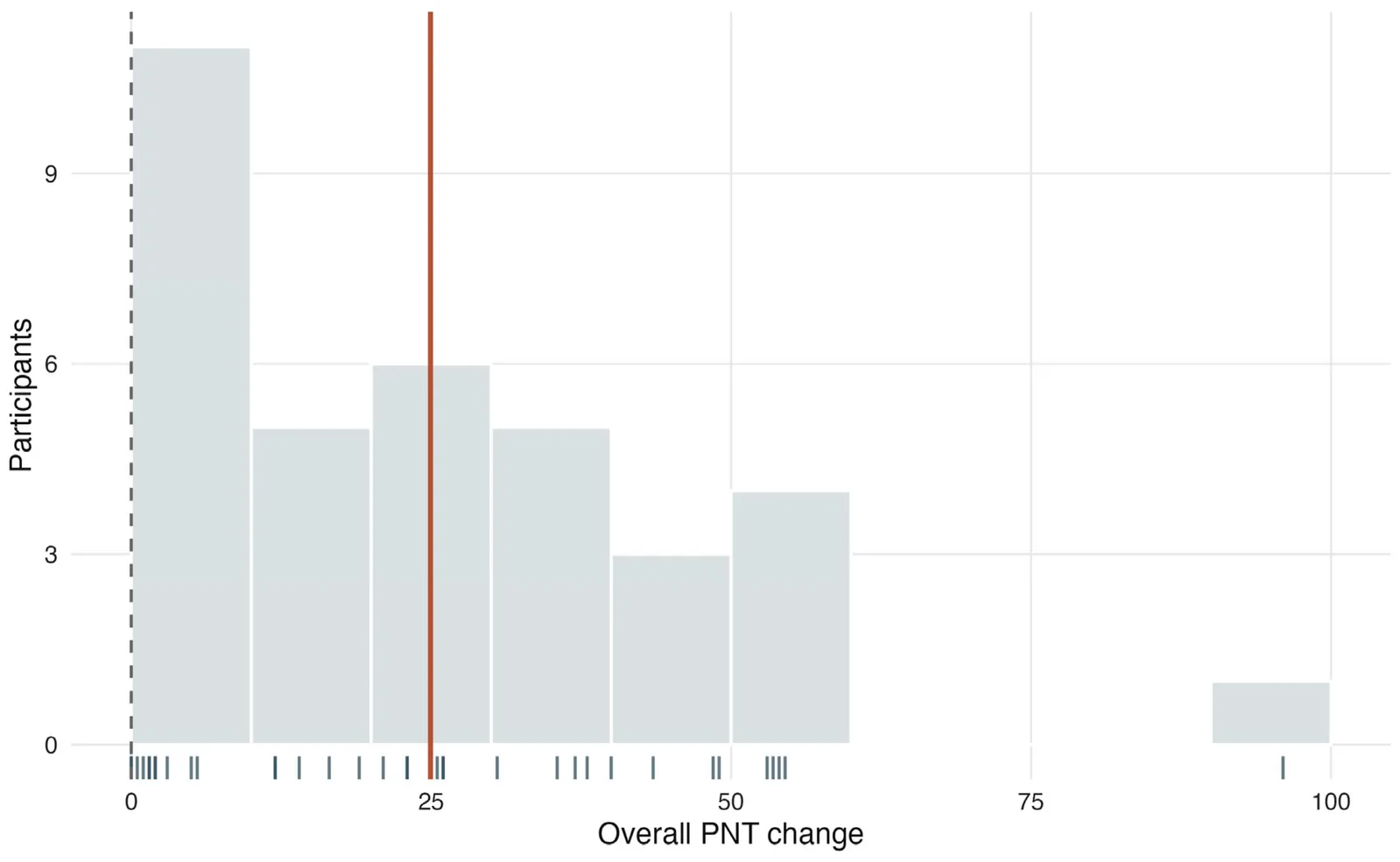
Distribution of overall PNT change, defined as last observed PNT minus first observed PNT. Histogram bars show participant counts in 10-point PNT-change bins; rug marks along the *x*-axis show individual participant values. The solid orange line indicates the sample mean.

Overall change in PNT score per participant was defined as the last observed PNT minus the first observed PNT. Across 35 participants, the mean overall change was 24.94 points (SD = 22.32). A total of 33 participants improved, 2 were unchanged, and none declined (Figure 5).

**Figure 6:**
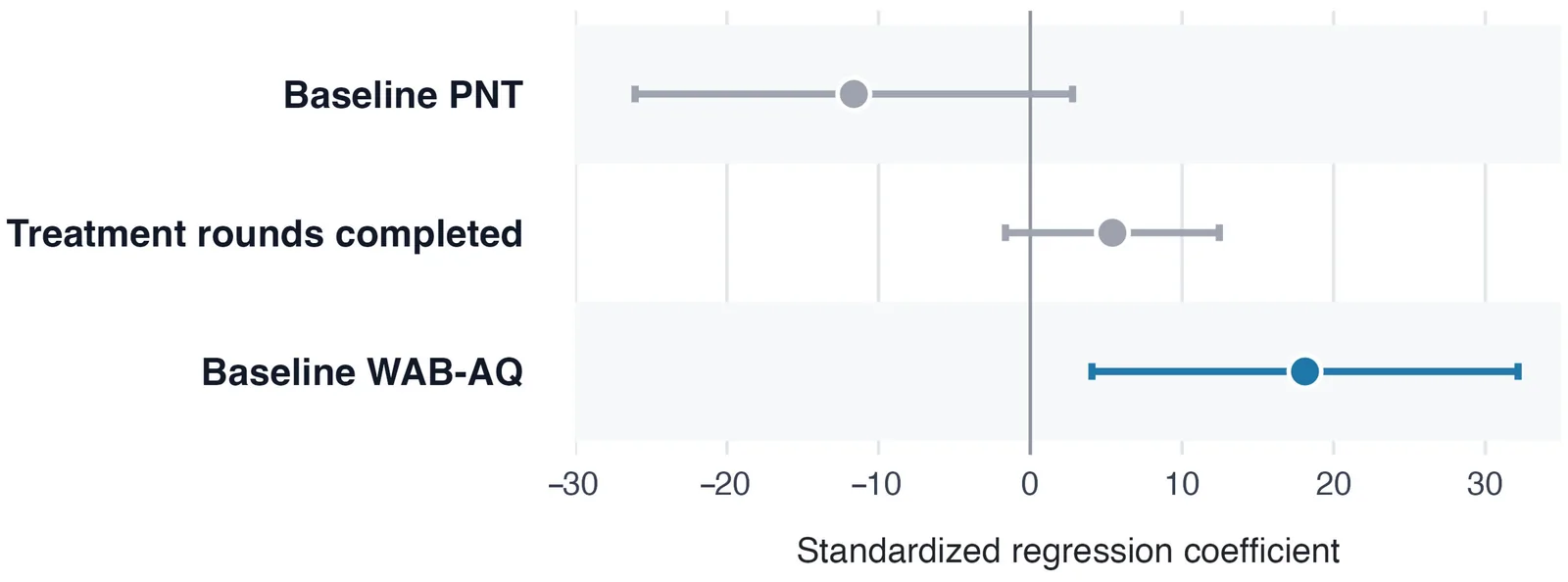
AICc-selected multivariable predictors of overall PNT change.

To examine factors associated with overall change in PNT scores, all candidate predictors were tested in exploratory univariate linear regression models (Supplementary Table S6). The only statistically significant association was WAB-AQ: higher baseline WAB-AQ was associated with greater overall PNT change (beta = 0.46, *p* = 0.024). Several additional variables showed trend-level associations, including number of treatment rounds completed (beta = 10.70, *p* = 0.141), age at stroke (beta = −0.52, *p* = 0.122), baseline PNT (beta = 0.11, *p* = 0.182), and aphasia type (*p* = 0.177), with lower overall change in the global/Wernicke group relative to Broca’s aphasia (beta = −19.29, *p* = 0.115). All-subsets AICc selection identified the strongest multivariable model as composed of baseline WAB-AQ (beta = 18.11, *p* = 0.013), number of treatment rounds completed (beta = 5.41, *p* = 0.127), and baseline PNT (beta = −11.64, *p* = 0.110). This model explained 27.8% of the variance in overall PNT change (Figure 6; Supplementary Table S7).

## 4. Discussion

We used retrospective data extracted from prior aphasia treatment studies to test whether repeated treatment exposure was associated with continued naming improvement in people with post-stroke aphasia. Specifically, we modeled longitudinal PNT change across successive treatment rounds among participants who completed at least two treatment studies. The primary mixed-effects model showed significant pre- to post-treatment gains in PNT performance across the first three treatment rounds, and the timepoint-by-treatment-round interaction was not significant. Thus, within this retrospective repeat-treatment sample, there was no statistical evidence that naming gains were attenuated in later treatment rounds relative to the first round. This finding directly addresses a central gap in the aphasia treatment literature: although chronic aphasia recovery is increasingly understood as dynamic rather than fixed, few studies have explicitly tested whether additional treatment exposure is associated with meaningful language gains after prior treatment participation.

The findings extend prior work on chronic aphasia recovery. Longitudinal studies have shown that language abilities can change years after stroke, and that chronic-stage aphasia trajectories include improvement, stability, and decline rather than a uniform plateau [13,15]. Earlier treatment reviews likewise argued that time post-onset alone is a poor basis for determining treatment candidacy [16]. The present study adds a treatment-specific contribution to this literature by showing that repeated treatment rounds, delivered across multiple source studies, were each associated with positive naming change. Sensitivity analyses supported this conclusion after adjustment for days post-stroke, alternative treatment-order coding, outlier exclusion, and random-effect structure. Taken together, these findings provide no evidence that treatment-associated naming gains were attenuated in later treatment rounds relative to earlier rounds.

The absence of observed attenuation across treatment rounds is clinically meaningful when considered in the context of the broader aphasia treatment literature. Meta-analytic, systematic-review, and randomized-trial evidence has established that SLT can improve language outcomes after stroke, including during the chronic stage [7,8,9,10,11], and treatment studies have long emphasized principles that are directly relevant to repeated treatment exposure: sufficient treatment intensity, repeated task-specific practice, communicative relevance, and opportunities for treatment-induced neuroplasticity [29,30,31,32,33]. Constraint-induced and intensive language-treatment approaches, for example, were developed partly around the premise that chronic aphasia can remain responsive to structured, high-dose language practice rather than becoming fixed after the early recovery window [30,31,32]. In parallel, participation-oriented frameworks have argued that aphasia rehabilitation should be responsive to changing life demands and should not be reduced to a single impairment-focused treatment episode [34]. The present findings add to this literature by suggesting that repeated treatment exposure can remain associated with naming gains even after prior treatment participation, which is consistent with a dynamic view of chronic aphasia recovery and argues against assuming that earlier therapy necessarily exhausts later treatment potential. At the same time, these data do not imply that all individuals will benefit equally from additional treatment or that treatment response is unconstrained; instead, they motivate prospective studies designed to test repeated-treatment effects directly and to identify which individuals are most likely to benefit from additional therapy within a personalized aphasia rehabilitation framework [12,13,17].

Two additional analyses support this interpretation without changing the primary inference. First, source-study analyses showed that PNT levels differed across studies, as expected given differences in recruitment, eligibility criteria, treatment mechanisms, and historical period, but source-study analyses did not account for the longitudinal treatment effect. Second, the interval-level analysis showed that naming improved more rapidly during treatment intervals than during the longer periods between treatments, suggesting that treatment periods were associated with a behavioral boost in naming performance. This pattern fits with aphasia treatment research emphasizing repeated, structured, and intensive language practice as a driver of treatment-related change [29,30,31,32,35], as well as broader rehabilitation principles that experience-dependent plasticity depends on repeated, behaviorally relevant practice over time [33,36]. In this sense, repeated treatment exposure may bolster recovery within a language system that remains dynamic rather than fixed, extending the dynamic recovery framework from the natural history of chronic aphasia to the treatment context [13,15].

The predictor analyses further connect the repeated-treatment findings to personalized aphasia rehabilitation. Baseline PNT and WAB-AQ were strong predictors of longitudinal PNT performance, and baseline WAB-AQ was the only statistically significant univariate predictor of overall PNT change. In the AICc-selected participant-level model, overall PNT change was best characterized by baseline WAB-AQ, number of treatment rounds completed, and baseline PNT. This pattern is consistent with prior work showing that aphasia treatment response is heterogeneous and that clinical, behavioral, and demographic variables can inform prognosis [12,17]. The negative adjusted coefficient for baseline PNT in the multivariable model should be interpreted cautiously in the context of baseline WAB-AQ and cumulative treatment exposure; it may reflect shared variance between baseline naming and overall aphasia severity, scale constraints, or different opportunities for measurable change across the severity continuum. Overall, these results support a personalized-treatment framework in which prognosis should account not only for response to an individual treatment course, but also for the likelihood of continued benefit across repeated treatment opportunities.

Taken together, the findings favor a broader agenda for studying aphasia rehabilitation as a longitudinal, adaptive process. Prospective repeated-treatment trials remain the clearest way to test causal effects of serial treatment exposure, but such studies are time-consuming, expensive, and difficult to sustain because they require repeated recruitment, treatment delivery, follow-up assessment, and participant retention over long intervals. The field should therefore pair prospective repeated-treatment designs with more scalable approaches. Pragmatic registries and harmonized multi-study datasets could capture real-world repeated treatment exposure across clinics and research programs; computerized and app-based therapy platforms could support high-frequency home practice while recording dose, adherence, and item-level performance [37,38]; telerehabilitation and hybrid-care models could reduce access barriers and make repeated treatment episodes more feasible over time [39,40]; and adaptive trial designs could test when, for whom, and under what conditions additional treatment should be intensified, maintained, or changed [41]. In parallel, computational neurorehabilitation and digital-twin-style recovery models offer a framework for integrating longitudinal behavioral, clinical, treatment-dose, and neurobiological data to simulate individualized recovery trajectories and optimize treatment decisions [42]. These approaches would move the field beyond asking whether aphasia treatment works on average and toward estimating how repeated treatment opportunities can be sequenced, personalized, and sustained to maximize long-term communication recovery.

### Limitations

Several limitations should guide interpretation. The study was retrospective and observational, and participants were included because they completed at least two treatment studies. This criterion likely selected for individuals who were willing and able to return for additional research participation, had access to repeated treatment opportunities, met study-specific eligibility criteria, and may have had positive prior treatment experiences. As a result, the findings generalize most directly to repeat treatment-study participants rather than to all individuals with post-stroke aphasia. The absence of random assignment to repeated treatment exposure or an untreated comparison condition also means that pre- to post-treatment change cannot be attributed uniquely to treatment. Practice effects, regression to the mean, spontaneous fluctuation, unmeasured clinical care, and broader life experience may have contributed to observed change.

The dataset was also unbalanced in ways that are inherent to retrospective repeated-treatment research. Study order, treatment type, participant characteristics (e.g., distribution of aphasia types, potential presence/severity of apraxia of speech), treatment dose, and historical period were not fully crossed, and later treatment rounds were sparse, with only one participant contributing a fourth round. Therefore, the present analyses cannot determine whether any single treatment was more effective than others, or whether specific participant profiles benefitted preferentially from specific treatment approaches. Source-study analyses and sensitivity models helped address these concerns, but they cannot remove selection bias or establish causal effects of cumulative treatment exposure. Available predictors were limited to demographic and clinical variables; lesion location, lesion volume, network integrity, treatment fidelity, detailed psycholinguistic profiles, and patient-centered outcomes were not modeled. Finally, the primary outcome was PNT naming performance, so the findings should not be generalized automatically to discourse, functional communication, auditory comprehension, reading, writing, participation, or quality of life. These limitations underscore the need for prospective and scalable longitudinal approaches that can test repeated treatment exposure while capturing broader outcomes and richer predictors of sustained response.

## 5. Conclusions

Using retrospective data from participants who completed multiple aphasia treatment studies, we found significant pre- to post-treatment gains in PNT performance across successive treatment rounds and no statistical evidence that treatment-associated naming gains were attenuated in later rounds. Interval-level analyses further suggested that naming improved more rapidly during treatment periods than during the longer intervals between treatments, supporting the interpretation that repeated treatment opportunities may provide a behavioral boost within an already dynamic recovery trajectory. Participant-level analyses indicated that baseline language status, particularly WAB-AQ, was associated with overall naming change, reinforcing the need for personalized models of sustained treatment response. Because the study was retrospective and observational, the findings should not be interpreted as definitive causal evidence that repeated treatment exposure produces continued recovery in all individuals. Rather, they support a longitudinal view of aphasia rehabilitation in which additional treatment opportunities can remain beneficial in the chronic stage and motivate prospective, scalable, and data-rich approaches to determine when, for whom, and how repeated treatment should be delivered.

## Figures and Tables

**Table 1:** Study sample descriptive statistics.

Variable	Value
Participants	35
Female, n (%)	8 (22.9%)
Male, n (%)	27 (77.1%)
Race	
Black, n (%)	8 (22.9%)
White, n (%)	27 (77.1%)
Stroke age in years, mean (SD)	56.0 (11.3)
Days post-stroke at first assessment, mean (SD)	1663.3 (1598.3)
Baseline PNT, mean (SD)	50.1 (47.3)
Baseline WAB-AQ, mean (SD)	51.1 (19.0)
Number of treatment studies completed	
Completed 2 studies, n (%)	25 (71.4%)
Completed 3 studies, n (%)	9 (25.7%)
Completed 4 studies, n (%)	1 (2.9%)

**Table 2:** Primary model estimated pre- to post-treatment PNT change by treatment round.

Treatment Round	Estimate	Standard Error	95% CI	df	*t*	*p*
1	3.86	1.36	[1.16, 6.56]	82.1	2.84	0.006
2	5.07	1.38	[2.33, 7.81]	82.3	3.68	<0.001
3	7.75	2.54	[2.70, 12.80]	82.1	3.05	0.003

**Table 3:** Clinical and demographic predictors of longitudinal PNT scores. Each covariate was individually entered into the primary linear mixed-effects model as a main effect and interaction with treatment rounds. Continuous variables were standardized; categorical variables were relative to the reference group. Abbreviations: B = black, F = female, M = male, W = white.

			Interaction with Treatment Round		
Covariate	Main Effect Estimate	*p*	2nd Round Estimate	3rd Round Estimate	*p*
Baseline PNT	46.72	<0.001	4.40	−3.65	0.168
Baseline WAB-AQ	38.64	<0.001	5.41	5.40	0.181
Sex (M vs. F)	32.38	0.112	−1.23	0.85	0.983
Age at stroke	−14.39	0.116	0.60	2.49	0.930
Days post-stroke	5.61	0.550	−0.79	−0.43	0.970
Race (W vs. B)	−1.73	0.710	3.56	−21.42	0.336
Aphasia type					
Anomic/conduction vs. Broca’s	27.48	0.012	13.68	−4.04	0.104
Global/Wernicke’s vs. Broca’s	−45.06	0.012	−6.41	Not estimated	

## Data Availability

The data presented in this study are available upon request to the corresponding author. The data are not publicly available due to privacy restrictions.

## Supplementary Materials

The following supporting information can be downloaded at: https://www.mdpi.com/article/10.3390/brainsci16090905/s1. Supplementary Table S1 Source study participation by treatment order, reports the number of participants completing each source study as their first, second, third, or fourth observed treatment study. Supplementary Figure S1 Distributions of candidate predictor variables, shows candidate predictor distributions. Supplementary Table S2 Descriptive PNT responder summaries by treatment round, reports descriptive responder summaries. Supplementary Table S3 Sensitivity analyses for the primary mixed-effects model, and Table S4 Exploratory equivalence tests comparing later-round gains with round 1 gains using a +/−5 PNT-point margin, report primary-model sensitivity and exploratory equivalence analyses. Supplementary Table S5 Exploratory equivalence tests comparing later-round gains with round 1 gains using a +/−5 PNT-point margin, reports the exploratory source-study-by-timepoint model, and Supplementary Figure S2 Source study-specific pre- to post-treatment PNT change, shows source-study-specific pre- to post-treatment PNT change. Supplementary Table S6 Source study-specific pre- to post-treatment PNT change, reports univariate participant-level predictors of overall PNT change, and Supplementary Table S7 AICc-selected multivariable model predicting overall PNT change, reports the AICc-selected multivariable model.

## References

[1] Flowers H.L., Skoretz S.A., Silver F.L., Rochon E., Fang J., Flamand-Roze C., Martino R. (2016). Poststroke aphasia frequency, recovery, and outcomes: A systematic review and meta-analysis. Arch. Phys. Med. Rehabil..

[2] Hilari K., Needle J.J., Harrison K.L. (2012). What are the important factors in health-related quality of life for people with aphasia? A systematic review. Arch. Phys. Med. Rehabil..

[3] Nickels L. (2002). Therapy for naming disorders: Revisiting, revising, and reviewing. Aphasiology.

[4] Roach A., Schwartz M.F., Martin N., Grewal R.S., Brecher A. (1996). The Philadelphia Naming Test: Scoring and rationale. Clin. Aphasiol..

[5] Fridriksson J., Hillis A.E. (2021). Current approaches to the treatment of post-stroke aphasia. J. Stroke.

[6] Fama M.E., Turkeltaub P.E. (2014). Treatment of poststroke aphasia: Current practice and new directions. Semin. Neurol..

[7] Robey R.R. (1998). A meta-analysis of clinical outcomes in the treatment of aphasia. J. Speech Lang. Hear. Res..

[8] Brady M.C., Kelly H., Godwin J., Enderby P., Campbell P. (2016). Speech and language therapy for aphasia following stroke. Cochrane Database Syst. Rev..

[9] Breitenstein C., Grewe T., Floel A., Ziegler W., Springer L., Martus P., Huber W., Willmes K., Ringelstein E.B., Haeusler K.G. (2017). Intensive speech and language therapy in patients with chronic aphasia after stroke: A randomised, open-label, blinded-endpoint, controlled trial in a health-care setting. Lancet.

[10] RELEASE Collaborators (2022). Dosage, intensity, and frequency of language therapy for aphasia: A systematic review–based, individual participant data network meta-analysis. Stroke.

[11] RELEASE Collaborators (2022). Precision rehabilitation for aphasia by patient age, sex, aphasia severity, and time since stroke? A prespecified, systematic review-based, individual participant data, network, subgroup meta-analysis. Int. J. Stroke.

[12] Kristinsson S., den Ouden D.B., Rorden C., Newman-Norlund R., Neils-Strunjas J., Fridriksson J. (2022). Predictors of therapy response in chronic aphasia: Building a foundation for personalized aphasia therapy. J. Stroke.

[13] Hope T.M.H., Leff A.P., Prejawa S., Bruce R., Haigh Z., Lim L., Ramsden S., Oberhuber M., Ludersdorfer P., Crinion J. (2017). Right hemisphere structural adaptation and changing language skills years after left hemisphere stroke. Brain.

[14] Aphasia United (2024). Best Practice Recommendations for Aphasia. https://www.aphasiaunited.org/best-practice-recommendations/.

[15] Holland A.L., Fromm D., Forbes M., MacWhinney B. (2017). Long-term recovery in stroke accompanied by aphasia: A reconsideration. Aphasiology.

[16] Moss A., Nicholas M. (2006). Language rehabilitation in chronic aphasia and time postonset: A review of single-subject data. Stroke.

[17] Kristinsson S., Basilakos A., Elm J., Spell L.A., Bonilha L., Rorden C., den Ouden D.B., Cassarly C., Sen S., Hillis A. (2021). Individualized response to semantic versus phonological aphasia therapies in stroke. Brain Commun..

[18] Fergadiotis G., Kellough S., Hula W.D. (2015). Item response theory modeling of the Philadelphia Naming Test. J. Speech Lang. Hear. Res..

[19] Fridriksson J., Hubbard H.I., Hudspeth S.G., Holland A.L., Bonilha L., Fromm D., Rorden C. (2012). Speech entrainment enables patients with Broca’s aphasia to produce fluent speech. Brain.

[20] Fridriksson J., Rorden C., Elm J., Sen S., George M.S., Bonilha L. (2018). Transcranial direct current stimulation vs sham stimulation to treat aphasia after stroke: A randomized clinical trial. JAMA Neurol..

[21] Bonilha L., Rorden C., Roth R., Sen S., George M.S., Fridriksson J. (2024). Improved naming in patients with Broca’s aphasia with tDCS. J. Neurol. Neurosurg. Psychiatry.

[22] Cassarly C., Doyle A., Ly T., Horn J., Aitchison M., Elm J., Fridriksson J., Bonilha L. (2021). Speech Entrainment for Aphasia Recovery (SpARc) phase II trial design. Contemp. Clin. Trials Commun..

[23] Spell L.A., Richardson J.D., Basilakos A., Stark B.C., Teklehaimanot A., Hillis A.E., Fridriksson J. (2020). Developing, implementing, and improving assessment and treatment fidelity in clinical aphasia research. Am. J. Speech Lang. Pathol..

[24] Kertesz A. (2007). The Western Aphasia Battery-Revised.

[25] Wallace S.J., Worrall L., Rose T., Le Dorze G., Breitenstein C., Hilari K., Babbitt E., Bose A., Brady M., Cherney L.R. (2019). A core outcome set for aphasia treatment research: The ROMA consensus statement. Int. J. Stroke.

[26] Goodglass H., Kaplan E., Barresi B. (2001). The Assessment of Aphasia and Related Disorders.

[27] Bates D., Maechler M., Bolker B., Walker S. (2015). Fitting linear mixed-effects models using lme4. J. Stat. Softw..

[28] Kuznetsova A., Brockhoff P.B., Christensen R.H.B. (2017). lmerTest package: Tests in linear mixed effects models. J. Stat. Softw..

[29] Bhogal S.K., Teasell R., Speechley M. (2003). Intensity of aphasia therapy, impact on recovery. Stroke.

[30] Pulvermuller F., Neininger B., Elbert T., Mohr B., Rockstroh B., Koebbel P., Taub E. (2001). Constraint-induced therapy of chronic aphasia after stroke. Stroke.

[31] Pulvermuller F., Berthier M.L. (2008). Aphasia therapy on a neuroscience basis. Aphasiology.

[32] Cherney L.R., Patterson J.P., Raymer A., Frymark T., Schooling T. (2008). Evidence-based systematic review: Effects of intensity of treatment and constraint-induced language therapy for individuals with stroke-induced aphasia. J. Speech Lang. Hear. Res..

[33] Kiran S., Thompson C.K. (2019). Neuroplasticity of language networks in aphasia: Advances, updates, and future challenges. Front. Neurol..

[34] Chapey R., Duchan J.F., Elman R.J., Garcia L.J., Kagan A., Lyon J.G., Simmons Mackie N. (2000). Life Participation Approach to Aphasia: A statement of values for the future. ASHA Lead..

[35] Basso A., Macis M. (2011). Therapy efficacy in chronic aphasia. Behav. Neurol..

[36] Kleim J.A., Jones T.A. (2008). Principles of experience-dependent neural plasticity: Implications for rehabilitation after brain damage. J. Speech Lang. Hear. Res..

[37] Palmer R., Dimairo M., Cooper C., Enderby P., Brady M., Bowen A., Latimer N., Julious S., Cross E., Alshreef A. (2019). Self-managed, computerised speech and language therapy for patients with chronic aphasia post-stroke compared with usual care or attention control (Big CACTUS): A multicentre, single-blinded, randomised controlled trial. Lancet Neurol..

[38] Des Roches C.A., Balachandran I., Ascenso E.M., Tripodis Y., Kiran S. (2015). Effectiveness of an impairment-based individualized rehabilitation program using an iPad-based software platform. Front. Hum. Neurosci..

[39] Laver K.E., Adey-Wakeling Z., Crotty M., Lannin N.A., George S., Sherrington C. (2020). Telerehabilitation services for stroke. Cochrane Database Syst. Rev..

[40] Cramer S.C., Dodakian L., Le V., See J., Augsburger R., McKenzie A., Zhou R.J., Chiu N.L., Heckhausen J., Cassidy J.M. (2019). Efficacy of home-based telerehabilitation vs in-clinic therapy for adults after stroke: A randomized clinical trial. JAMA Neurol..

[41] Lei H., Nahum-Shani I., Lynch K., Oslin D., Murphy S.A. (2012). A SMART design for building individualized treatment sequences. Annu. Rev. Clin. Psychol..

[42] Reinkensmeyer D.J., Burdet E., Casadio M., Krakauer J.W., Kwakkel G., Lang C.E., Swinnen S.P., Ward N.S., Schweighofer N. (2016). Computational neurorehabilitation: Modeling plasticity and learning to predict recovery. J. Neuroeng. Rehabil..

