# Supplementary material for: Beyond the Plateau: Repeated Treatment Opportunities Are Associated with Improved Naming in Chronic Post-Stroke Aphasia
Source: Brain Sci. 2026 Aug 25;16(9):905. doi: 10.3390/brainsci16090905 (PMC13604328; doi:10.3390/brainsci16090905)
Supplement: Supplementary file 1 [file brainsci-16-00905-s001.zip › brainsci-4468219-supplementary.pdf]

Supplementary Materials

Beyond the Plateau: Repeated Treatment Opportunities Are Associated with Improved Naming in Chronic Post-Stroke Aphasia

Supplementary Table S1. Source study participation by treatment order, reports the number of participants completing each source study as their first, second, third, or fourth observed treatment study.

Source study	1st round	2nd round	3rd round	4th round	Total
CT	22	0	1	0	23
POLAR	9	20	5	0	34
R01SE	4	8	0	0	12
SPARC	0	7	4	1	12

Supplementary Figure S1. Distributions of candidate predictor variables, shows candidate predictor distributions.

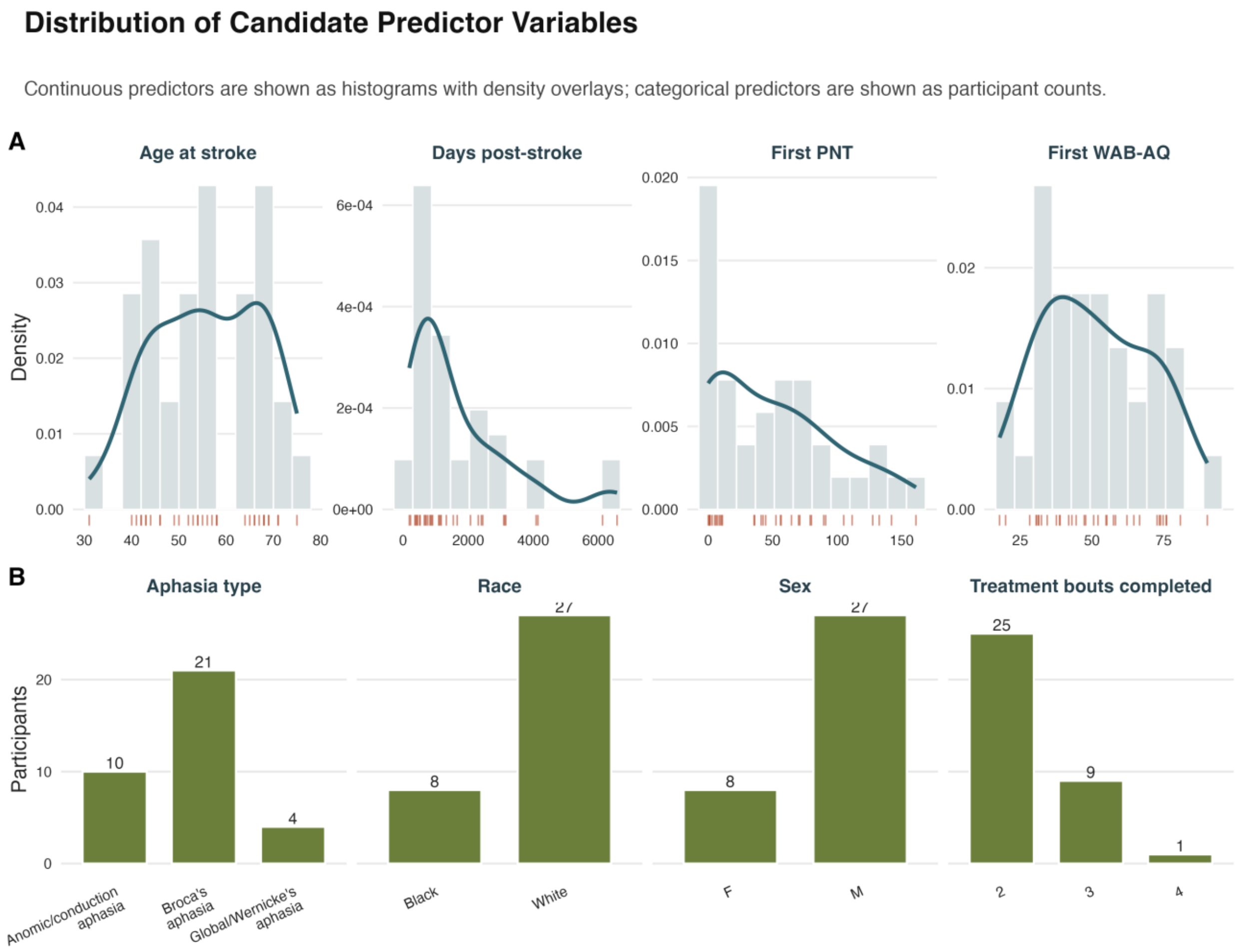

Supplementary Table S2. Descriptive PNT responder summaries by treatment round, reports descriptive responder summaries.

Round	n	> 0 points	>= 5 points	>= 10 points	Unchanged	Declined
1	35	17 (48.6%)	11 (31.4%)	9 (25.7%)	7	11
2	34	27 (79.4%)	14 (41.2%)	8 (23.5%)	2	5
3	10	8 (80.0%)	5 (50.0%)	4 (40.0%)	0	2

Supplementary Table S3. Sensitivity analyses for the primary mixed-effects model.

Model	n obs.	n participants	Estimated gain	Timepoint p	Interaction p	Note
Primary model	159	35	5.56	< .001	.384	Categorical treatment bout/order
Adjusted for assessment days post-stroke	159	35	5.23	< .001	.421	Adds visit-level days post-stroke
Treatment bout/order modeled ordinally	159	35	4.87	< .001	.184	TxBout modeled as centered/scaled ordinal variable
Excluding IQR outlier participant(s)	155	34	5.49	< .001	.319	Excluded M2049
Participant random slope for timepoint	159	35	5.53	< .001	.378	Includes participant-specific timepoint slope

Supplementary Table S4. Exploratory equivalence tests comparing later-round gains with round 1 gains using a +/-5 PNT-point margin, report primary-model sensitivity and exploratory equivalence analyses.

Contrast	Estimate	95% CI	TOST p	Equivalent
Bout 2 gain - Bout 1 gain	1.21	[-2.63, 5.06]	.027	Yes
Bout 3 gain - Bout 1 gain	3.89	[-1.83, 9.62]	.351	No

Supplementary Table S5. Exploratory equivalence tests comparing later-round gains with round 1 gains using a +/-5 PNT-point margin, reports the exploratory source-study-by-timepoint model.

Effect	df1	df2	F	p
Timepoint	1.00	79.26	21.17	< .001
TxBoutF	2.00	47.57	3.40	.042
Study	3.00	47.19	3.30	.028
Timepoint:TxBoutF	2.00	79.15	0.67	.514
Timepoint:Study	3.00	79.26	0.74	.529

Supplementary Figure S2. Source study-specific pre- to post-treatment PNT change, shows source-study-specific pre- to post-treatment PNT change.

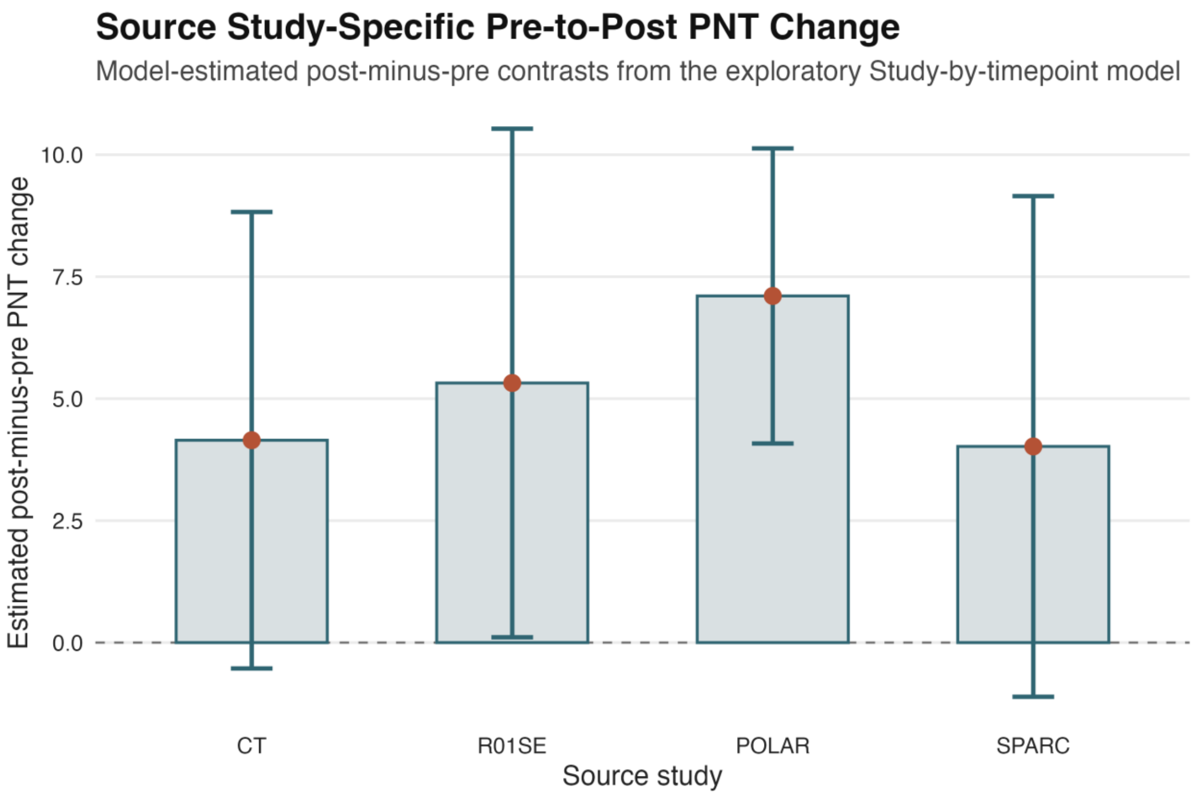

Supplementary Table S6. Source study-specific pre- to post-treatment PNT change, reports univariate participant-level predictors of overall PNT change.

Predictor	Coefficient/contrast	n	Beta	SE	t	p	R2
First WAB-AQ	Per WAB-AQ point	34	0.46	0.19	2.37	.024	0.15
Aphasia type	Global/Wernicke's vs Broca's	35	-19.29	11.89	-1.62	.115	0.10

Age at stroke	Per year	35	-0.52	0.33	-1.59	.122	0.07
Treatment bouts completed	Per additional bout	35	10.70	7.09	1.51	.141	0.06
First PNT	Per PNT point	35	0.11	0.08	1.36	.182	0.05
Sex	Male vs female	35	6.16	9.06	0.68	.501	0.01
Aphasia type	Anomic/conduction vs Broca's	35	5.18	8.38	0.62	.540	0.10
Days post-stroke	Per day	35	0.00	0.00	0.49	.624	0.01
Race	White vs Black	35	3.17	9.10	0.35	.730	0.00

Supplementary Table S7. AICc-selected multivariable model predicting overall PNT change, reports the AICc-selected multivariable model.

Predictor	Beta	SE	95% CI	t	p
Number of treatment rounds completed, z	5.41	3.45	[-1.64, 12.47]	1.57	.127
Baseline WAB-AQ, z	18.11	6.88	[4.06, 32.16]	2.63	.013
Baseline PNT, z	-11.64	7.06	[-26.06, 2.79]	-1.65	.110
